# Patient-reported outcomes in patients with cystic fibrosis with a *G551D* mutation on ivacaftor treatment: results from a cross-sectional study

**DOI:** 10.1186/s12890-019-0887-6

**Published:** 2019-08-13

**Authors:** Scott C. Bell, Jochen G. Mainz, Gordon MacGregor, Susan Madge, Julie Macey, Moshe Fridman, Ellison D. Suthoff, Siva Narayanan, Nils Kinnman

**Affiliations:** 1 Department of Thoracic Medicine, The Prince Charles Hospital and QIMR Berghofer Medical Research Institute, Brisbane, QLD Australia; 2 Jena University Hospital, Jena, Germany; 3 Brandenburg Medical School (MHB), University , Brandenburg an der Havel, Germany; 4 Gartnavel General Hospital, Glasgow, UK; 5 Royal Brompton Hospital, London, UK; 6 University Hospital Bordeaux, Bordeaux, France; 7 AMF Consulting, Los Angeles, CA USA; 8 Formerly of Vertex Pharmaceuticals Incorporated, Boston, MA USA; 9 Decision Resources Group, Burlington, MA USA; 10 Vertex Pharmaceuticals Incorporated, Boston, MA USA

**Keywords:** Patient-reported outcomes, Work productivity, Ivacaftor, Cystic fibrosis, *G551D-CFTR*

## Abstract

**Background:**

Clinical studies demonstrate that ivacaftor (IVA) improves health-related quality of life (HRQoL) in patients aged ≥6 years with cystic fibrosis (CF). The real-world impact of IVA and standard of care (SOC) in groups of patients with *G551D* and *F508del* mutations, respectively, was assessed using a survey comprising disease-specific and generic HRQoL measures.

**Methods:**

Patients with CF aged ≥12 years, or aged 6–11 years with caregiver support, with either (1) a *G551D* mutation and receiving IVA (*G551D*/IVA) for ≥3 months, or (2) homozygous for *F508del* and receiving SOC before lumacaftor/IVA availability (*F508del*/SOC), were eligible to participate in a cross-sectional survey. Demographic and clinical characteristics, and HRQoL measures were compared between patient groups, and multiple regression analyses were conducted.

**Results:**

After differences in patient demographic and clinical characteristics were controlled for, significantly better scores were observed in the *G551D*/IVA group than in the *F508del*/SOC group on multiple domains of the validated Cystic Fibrosis Questionnaire-Revised and the EuroQol 5-dimensions 5-level questionnaire.

**Conclusions:**

*G551D*/IVA patients reported better HRQoL than *F508del*/SOC patients on generic and disease-specific measures in a real-world setting.

## Background

Cystic fibrosis (CF) is a chronic, progressive, life-shortening disease that affects the ability of patients to function in many areas of daily living [1, 2]. There is growing evidence that patient-reported outcomes (PROs) can serve as valuable indicators of the benefit of treatment and its impact on health-related quality of life (HRQoL), as they represent direct measures of how a patient feels and functions [3, 4]. Evaluation of such measures is becoming increasingly important for patients with chronic disease, such as CF, which necessitates complex management regimens and imposes a considerable burden on patients and their caregivers [1, 5, 6].

The Cystic Fibrosis Questionnaire-Revised (CFQ-R) is a disease-specific, validated tool that encompasses general domains of HRQoL as well as domains specific to CF [7, 8]. The EuroQol 5-dimensions 5-level questionnaire (EQ-5D-5L) is a generic measure that provides a single index value of health status and permits comparisons between treatments across different medical conditions [4, 9, 10]. The EQ-5D-5L is a widely accepted measure for assessing utilities, which is recommended by the National Institute for Health and Care Excellence in the United Kingdom (UK), and is a standardized assessment that can be used across a variety of diseases [11]. Other PROs, such as the Work Productivity and Activity Impairment Questionnaire (WPAI) [12, 13], take productivity losses into consideration, which may provide additional context for the impact of treatment on patients and their caregivers in the real world.

The treatment benefit of ivacaftor (IVA) in patients with CF with a *G551D* mutation in the cystic fibrosis transmembrane conductance regulator (*CFTR*) gene has been established in Phase 3 studies [14–16]. Improvements in lung function, body weight, and HRQoL, as measured by the CFQ-R Respiratory Symptoms domain, were observed through 48 weeks [14, 15]; these benefits were sustained for nearly 3 years in an open-label extension study [16]. An additional analysis using Phase 3 data further demonstrated broad HRQoL benefits of IVA across multiple domains of the CFQ-R [17]. However, the majority of HRQoL data with IVA have been derived from clinical trials [14–17], whereas HRQoL data from real-world studies are limited [18]. We therefore sought to expand on these studies by determining the impact of IVA on HRQoL using the CFQ-R and EQ-5D-5L, and work productivity using the WPAI. We also sought to compare outcomes for patients with the *G551D-CFTR* mutation on ≥1 allele who were receiving treatment with IVA (*G551D*/IVA group) in the real-world setting with those for patients homozygous for the *F508del-CFTR* mutation who were receiving standard of care (SOC) (*F508del*/SOC group) treatment prior to availability of lumacaftor (LUM)/IVA.

## Methods

Patients with CF who were ≥12 years of age and caregivers of patients aged 6–11 years were recruited to participate in this cross-sectional, observational study in 5 countries (France, the UK, Germany, Australia, and Ireland). The final study report date was October 2, 2014. Patients were screened by the site coordinators at the time of clinic visits to confirm study eligibility and to collect demographic and clinical characteristics. Patients who were eligible for the study were diagnosed with CF, had ≥1 *G551D* mutation, and were receiving IVA with treatment exposure for ≥3 months, or were homozygous for the *F508del* mutation and receiving SOC (not exposed to or receiving IVA and prior to the availability of LUM/IVA). Patients were excluded from the study if they were participating in an interventional clinical trial, experiencing a pulmonary exacerbation at their clinic visit, or unable or unwilling to provide consent (adults/caregivers) or assent (minors). A study screening form, completed by clinic staff, reported demographic and clinical patient data, such as percent predicted forced expiratory volume in 1 s (ppFEV_1_), height, weight, *CFTR* mutations, select key comorbidities (i.e. presence of anxiety, attention deficit hyperactivity disorder, depression, diabetes, cancer, infertility, end-stage liver disease, pulmonary hypertension, osteoporosis, pancreatic insufficiency, pancreatitis, chronic rhinosinusitis/nasal polyps, and rectal prolapse), and current CF treatment including IVA.

Patients or their caregivers who qualified and consented to participate in the study completed a one-time survey at the time of their clinic visit comprising the CFQ-R [7, 8], EQ-5D-5 L [9, 10], and WPAI [12, 13]. Three distinct age-specific versions of the survey were used for patients aged 6–11 years, 12–13 years, and ≥14 years. The survey was completed by the patients themselves with the following exceptions: patients aged 6–11 years (caregivers completed the survey) and patients aged ≥12 years (completed by the patient, with some assistance from the caregiver, as appropriate).

The CFQ-R measures HRQoL in general domains of Vitality, Health Perceptions, Physical Functioning, Emotional Functioning, Social Functioning, and Role Functioning, and CF-specific domains of Body Image, Eating, Treatment Burden, and Respiratory and Digestive Symptoms [7, 8]. The EQ-5D-5L has a descriptive system that measures general health status in 5 dimensions: Mobility, Self-Care, Usual Activities, Pain/Discomfort, and Anxiety/Depression, and a visual analog scale (VAS) that records an individual’s self-rated health on a scale of 0–100 [10]. Both the CFQ-R and EQ-5D-5L have been validated in CF [7, 8, 10]. The WPAI is designed to measure the effect of general health and symptom severity on work/school productivity and regular activities during the past 7 days; it has been validated in respiratory disease, but not in CF [12, 13].

Demographic and clinical characteristics of the *G551D*/IVA and *F508del*/SOC patient groups were compared, with *t*-tests for numeric variables and χ^2^ tests for categorical variables. Multivariate regression analyses were conducted for outcomes on the CFQ-R, EQ-5D-5L, and WPAI to compare *G551D/*IVA and *F508del/*SOC groups, adjusting for differences observed between groups in sex, ppFEV_1_, and number of comorbidities. Multiplicity was not controlled for. Least squares (LS) estimated means were presented as adjusted mean values. *P* values < 0.05 were considered statistically significant in all analyses.

Central ethics approval of the study protocol was obtained in the UK and France, while institution-specific ethics approval was obtained at each of the participating institutions from Germany, Australia, and Ireland.

## Results

Demographic and clinical characteristics of 209 survey respondents (72 in the *G551D*/IVA group and 137 in the *F508del*/SOC group) at study entry are shown in Table 1. In the *G551D*/IVA group, of the 72 surveys, 60 were completed by patients aged ≥12 years and 12 by caregivers for children aged 6–11 years. In the *F508del*/SOC group, of the 137 surveys, 116 were completed by patients aged ≥12 years and 21 by caregivers for children aged 6–11 years. Overall, the mean (standard deviation [SD]) patient age was 24.3 (12.1) years and 56% of patients were male (Table 1). Some differences were observed between groups. Age was similar between the two groups but the *G551D*/IVA group had a significantly higher proportion of females than the *F508del*/SOC group (60.3% vs. 35.2%, respectively; *P* <  0.01). The *G551D/*IVA group also had a significantly higher mean ppFEV_1_ than the *F508del*/SOC group (79.8 vs. 70.7 percentage points, respectively; *P* <  0.05), significantly fewer patients with pancreatic insufficiency (80.3% vs. 92.0%; *P* <  0.05), significantly fewer mean number of comorbidities (1.5 vs. 2.0; *P* <  0.01), and significantly lower proportion of patients with ≥1 comorbidity (84.5% vs. 96.4%; *P* <  0.01) (Table 1). Individual comorbidities for which significant differences were observed between *G551D*/IVA and *F508del*/SOC groups included pancreatitis (2.8% vs. 0.0%; *P* <  0.05) and nasal polyp formation (8.5% vs. 19.0%; *P* <  0.05). Body mass index (BMI) for those aged ≥19 years was similar between the groups, as was BMI z-score for those aged <19 years.

**Table 1:** Demographic and clinical characteristics

Characteristic	Overall (*N* = 209)	*G551D*/IVA patient group (*n* = 72)	*F508del*/SOC patient group (*n* = 137)	*P* value^a^
Age, mean (SD), years	24.3 (12.1)	23.9 (13.9)	24.6 (11.1)	0.70
Range	6–62	6–62	6–52	
Missing, *n*	6	3	3	
Age group (years), *n* (%)				
6–11	33 (15.8)	12 (16.7)	21 (15.3)	
12–17	39 (18.7)	18 (25.0)	21 (15.3)	0.19
≥18	137 (65.6)	42 (58.3)	95 (69.3)	
Sex, *n* (%)^b^				
Male	108 (56.0)	27 (39.7)	81 (64.8)	< 0.01
Female	85 (44.0)	41 (60.3)	44 (35.2)	
Missing, *n*	16	3	12	
ppFEV_1_ (%), mean (SD)	73.9^c^ (28.0)	79.8 (25.6)	70.7^d^ (28.8)	< 0.05
ppFEV_1_ <70, *n* (%)	85 (40.7)^e^	24 (33.3)	61 (44.5)^e^	0.12
ppFEV_1_ (%), *n*; mean (SD)				
<40	32; 29.3 (6.6)	6; 28.8 (4.5)	26; 29.3 (7.0)	0.87
40–69	51; 54.5 (8.7)	18; 57.4 (7.9)	33; 52.9 (8.7)	0.07
70–89	50; 79.2 (6.0)	16; 78.6 (6.6)	33; 79.5 (5.7)	0.60
≥90	74; 103.0 (10.5)	31; 103.4 (9.7)	43; 102.6 (11.1)	0.74
Number of comorbidities, mean (SD)	1.9^f^ (1.2)	1.5^g^ (1.1)	2.0 (1.2)	< 0.01
Patients with ≥1 comorbidity, *n* (%)	192 (92.3)	60 (84.5)	132 (96.4)	< 0.01
History of lung transplant, *n* (%)				
Yes	3^f^ (1.4)	0^g^ (0.0)	3 (2.2)	0.55
No	205^f^ (98.6)	71^g^ (100.0)	134 (97.8)	
Waiting for lung transplant, *n* (%)				
Yes	3^f^ (1.4)	0^g^ (0.0)	3 (2.2)	0.55
No	205^f^ (98.6)	71^g^ (100.0)	134 (97.8)	
BMI				
Aged ≥19 years, *n*; mean (SD), kg/m^2^	126; 21.6 (3.3)	36; 22.2 (3.2)	90; 21.3 (3.3)	0.16
Aged <19 years, *n*; mean (SD) z-score	59; 0.001 (0.87)	25; 0.004 (0.82)	34; −0.0009 (0.92)	0.98

*BMI* Body mass index, *IVA* Ivacaftor, *ppFEV*_*1*_ Percent predicted forced expiratory volume in 1 s, *SD* Standard deviation, *SOC* Standard of care

^a^*P* values presented are for comparison of the *G551D*/IVA group with the *F508del*/SOC group

^b^Percentages are derived from the available sample

^c^*n*=207

^d^*n*=135

^e^Baseline data was missing for 2 patients

^f^*n*=208

^g^*n*=71

The mean (SD) duration of IVA exposure was 21.8 (15.1) months. Patient recruitment and duration of IVA exposure varied by country, as shown in Table 2.

**Table 2:** Patient cohorts by country with duration of IVA exposure

Country	Overall	*G551D*/IVA patient group	*F508del*/SOC patient group
All countries, *N*	209	72^a^	137
IVA exposure, mean (SD), months	NA	21.8 (15.1)	NA
France, *n* (%)	61 (29.2)	10 (13.9)	51 (37.2)
IVA exposure, mean (SD), months	NA	15.9 (8.4)	NA
United Kingdom, *n* (%)	54 (25.8)	33 (45.8)	21 (15.3)
IVA exposure, mean (SD), months	NA	18.1^b^ (11.4)	NA
Germany, *n* (%)	47 (22.5)	14 (19.4)	33 (24.1)
IVA exposure, mean (SD), months	NA	19.6 (9.6)	NA
Australia, *n* (%)	38 (18.2)	12 (16.7)	26 (19.0)
IVA exposure, mean (SD), months	NA	35.6 (24.1)	NA
Ireland, *n* (%)	9 (4.3)	3 (4.2)	6 (4.4)
IVA exposure, mean (SD), months	NA	30.0 (0.0)	NA

*IVA* Ivacaftor, *NA* Not applicable, *SD* Standard deviation, *SOC* Standard of care

^a^Duration on ivacaftor exposure was missing for 6 patients

^b^*n* = 27

**Fig. 1:**
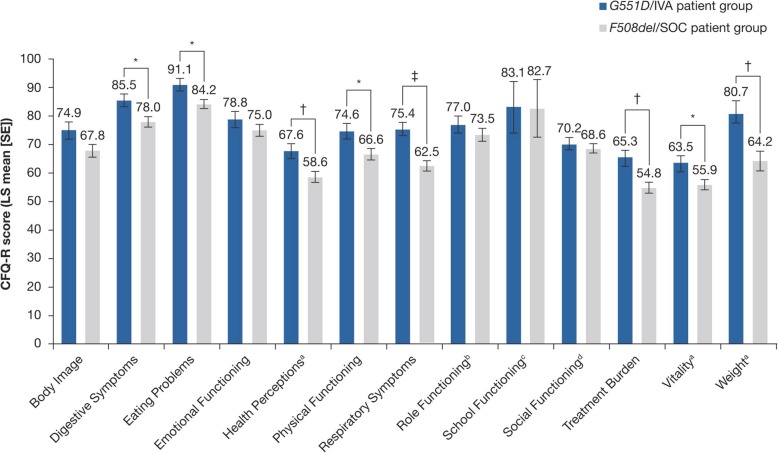
CFQ-R results by domain. The LS mean (SE) CFQ-R score for each domain is shown. *P < 0.05, ^†^*P* < 0.01, ^‡^*P* < 0.001 for the difference between *G551D*/IVA and *F508del*/SOC groups. ^a^ 6–11-year and ≥14-year versions only. ^b^ ≥14-year version only. ^c^ 6–11-year version only. ^d^12–13-year and ≥14-year versions only. *CFQ-R* Cystic Fibrosis Questionnaire-Revised, *IVA* ivacaftor, *LS* least squares, *SE* standard error, *SOC* standard of care

**Fig. 2:**
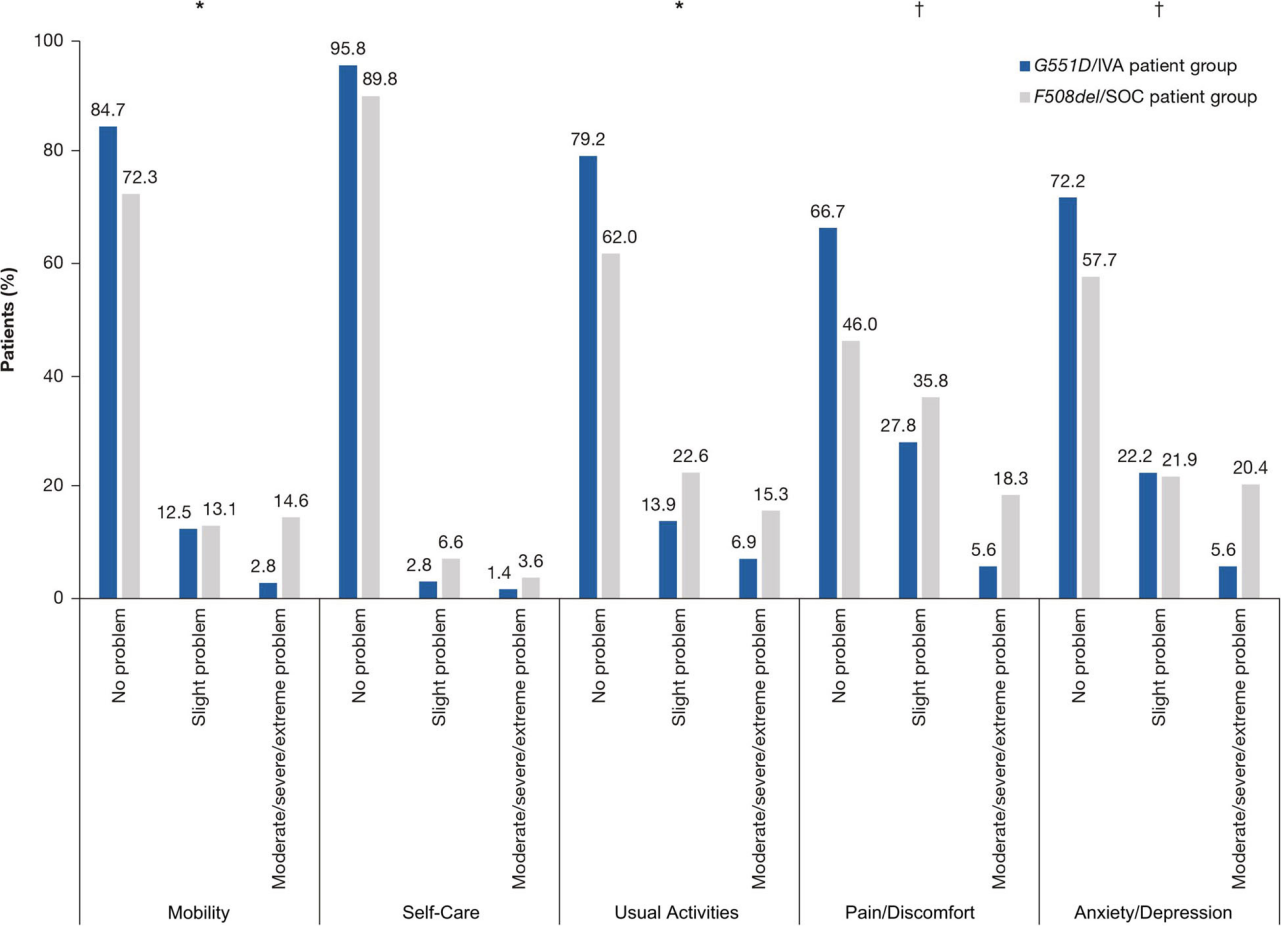
EQ-5D-5L results by domain. *P < 0.05, ^†^*P* < 0.01 for the difference in LS means between *G551D*/IVA and *F508del*/SOC groups. *EQ-5D-5L* EuroQol 5-dimensions 5-level questionnaire, *IVA* ivacaftor, *SOC* standard of care

Using an analysis of covariance model controlled for differences in ppFEV_1_, sex, and number of comorbidities at the time of their clinic visit, patients in the *G551D*/IVA group had significantly higher LS mean CFQ-R scores than patients in the *F508del*/SOC group in the following scales: Respiratory Symptoms (75.4 vs. 62.5; *P* < 0.0001), Digestive Symptoms (85.5 vs. 78.0; *P* < 0.05), Eating (91.1 vs. 84.2; *P* < 0.05), Treatment Burden (65.3 vs. 54.8; *P* < 0.01), and Physical Functioning (74.6 vs. 66.6; *P* < 0.05) (Fig. 1). Patients in the *G551D*/IVA group also had significantly higher LS mean CFQ-R scores than patients in the *F508del*/SOC group in the 6–11-year and the ≥14-year versions of the Weight (80.7 vs. 64.2; *P* < 0.01), Health Perceptions (67.6 vs. 58.6; *P* < 0.01), and Vitality (63.5 vs. 55.9; *P* < 0.05) (Fig. 1) domains. For the EQ-5D-5L, significantly better LS mean index scores and VAS scores were also observed in the *G551D*/IVA group than in the *F508del*/SOC group after controlling for differences in ppFEV_1_, sex, and number of comorbidities (Table 3). Within individual domains of the EQ-5D-5L, significantly better scores of Mobility, Usual Activities, Pain/Discomfort, and Anxiety/Depression were observed for the *G551D*/IVA group, whereas Self-Care was not significantly different between the groups (Fig. 2). In adjusted analyses of the WPAI, LS mean scores for School Productivity Loss and Daily Activity Impairment were numerically lower in the *G551D*/IVA group than in the *F508del*/SOC group, although differences between groups did not reach statistical significance (Table 3).

**Table 3:** EQ-5D-5L and WPAI results

	*G551D*/IVA patient group	*F508del*/SOC patient group	*P* value
EQ-5D-5L			
Index score (0–1), *n*; LS mean (SE)	72; 0.90 (0.02)	137; 0.81 (0.02)	< 0.01
VAS score (0–100), *n*; LS mean (SE)	72; 75.7 (1.8)	135; 70.0 (1.4)	0.0136
WPAI			
School, *n*^a^	41	53	
Productivity loss, *n*; LS mean (SE)^b^	27; 24.62 (6.69)	32; 34.57 (6.73)	0.3242
Daily activities, *n*	70	135	
Activity impairment, LS mean (SE)^c^	21.63 (2.95)	28.30 (2.19)	0.08

*CF* Cystic fibrosis, *EQ-5D-5L* EuroQol 5-dimensions 5-level questionnaire, *IVA* Ivacaftor, *LS* Least squares, *SE* Standard error, *SOC* Standard of care, *VAS* Visual analog scale, *WPAI* Work Productivity Activity and Impairment Questionnaire

^a^A total of 29 students were excluded because they were not in school over the past 2 weeks for reasons other than CF or had missing data

^b^Productivity loss: combination of absenteeism and presenteeism representing the percentage of overall impairment due to CF

^c^Activity impairment: percentage impact of CF on daily activities

## Discussion

This cross-sectional observational study demonstrated that treatment with IVA in patients with CF and a *G551D* mutation was associated with multiple HRQoL benefits on disease-specific and generic and validated PRO measures compared with patients with CF who were homozygous for *F508del* and receiving SOC. This study was designed to evaluate outcomes following IVA exposure for a longer period of time than the usual follow-up period of randomized controlled trials, with the mean duration of IVA exposure being 21.8 months. Better outcomes on the CFQ-R in multiple domains and on the EQ-5D-5L using both index and VAS scores were observed in the *G551D*/IVA group. These findings lend additional real-world support to the HRQoL benefit of IVA previously described in clinical trials [14–17].

In recent years, there has been mounting interest from regulatory authorities as well as funding authorities to evaluate HRQoL, efficacy, and safety in real-world settings, necessitating continued demonstration of health outcomes. In particular, measures that can generate a single index value to allow for comparisons across a disease state that contribute to cost analysis are needed [4]. Data on EQ-5D-5L scores are instrumental in health technology assessments (HTA) in several countries around the world [11]. In this analysis, significant differences between the *G551D*/IVA and *F508del*/SOC groups were observed on the EQ-5D-5L using both the index score and VAS. Although a minimum clinically important difference has not yet been established in CF, in chronic obstructive pulmonary disease, the mean estimates of the minimum clinically important difference for the utility index and EuroQol-VAS have been estimated at 0.051 (range 0.037–0.063) and 6.9 (range 6.5–8.0), respectively [19]. This is in line with the difference of 0.09 observed in this study. It should be noted that children with CF and their parents tend to develop coping mechanisms early in life and often establish a ‘new normal’ in their minds. This is sometimes reflected in high EQ-5D-5L scores, as seen in this study, and can make it difficult to identify differences in treatment effects using the EQ-5D-5L. Despite this, the statistically significant difference in the tool measures shown here mark a notable finding in this area.

With respect to the subscales on the EQ-5D-5L, we saw better scores for Mobility, Usual Activities, Pain/Discomfort, and Anxiety/Depression in the *G551D*/IVA group than in the *F508del*/SOC group. Although no difference in Self-Care was seen, previous observations in CF have also showed little impact on this scale [4, 10]. However, Pain/Discomfort and Anxiety/Depression scales have consistently been found to be highly impacted among patients with CF [4, 10]. These scales may be particularly informative in CF and warrant further study. Depression and anxiety have been reported at higher rates among patients with CF and their caregivers than in the general community [20]. Psychological symptoms in patients with CF have been associated with lower lung function, worse nutritional status, poorer adherence, lower HRQoL, and more frequent hospitalization [21]. Thus, the Cystic Fibrosis Foundation and the European Cystic Fibrosis Society developed a consensus statement for screening and treating depression and anxiety, recommending use of the Patient Health Questionnaire 9- and Generalized Anxiety Disorder 7-item scales for annual screening [21]. In our study, between-group differences were not significant on psychosocial domains of the CFQ-R questionnaire, suggesting that the CFQ-R questionnaire is less capable of capturing the psychosocial benefits of therapy with CFTR modulators. The role of pain in CF is less well studied, but recent reports indicate that pain is associated with depression, anxiety, lower HRQoL, and increased risk of pulmonary exacerbations and death [22, 23] and could relate to a decrease in the patient’s ability to participate in airway clearance activities, or pain could be a potential marker of increased inflammation and/or disease severity. More research is needed to evaluate pain and its effect on HRQoL and other health parameters, as well as to further assess pain management strategies [22, 23].

The benefits associated with IVA on the CFQ-R after a mean (SD) exposure of 21.8 (15.1) months were generally consistent with those observed using data from the Phase 3 STRIVE study [17]. Benefits on the Respiratory Symptoms domain were notable; a nearly 13-point difference was found between the *G551D*/IVA and *F508del*/SOC groups in adjusted analyses, exceeding the minimum clinically important difference of 4 points for the Respiratory Symptoms domain of the CFQ-R [24]. Furthermore, these data are consistent with the observational GOAL study, in which real-world patients with *G551D* mutations treated with IVA for 6 months showed a 7.4-point improvement from baseline in the CFQ-R Respiratory Symptoms domain [18].

Benefits on other CF-specific scales, including Weight, Digestive Symptoms, Eating, and Treatment Burden, were also observed. The between-group difference in Weight score of 16.5 points was particularly notable. Higher scores on general health domains of Physical Functioning, Health Perception, and Vitality were seen, although we did not see higher scores in Social Functioning, as seen in Phase 3 clinical trials [17].

In chronic diseases such as CF, lost productivity can contribute to the overall burden of disease. Additionally, work productivity data are considered in HTAs in several countries, and are considered a relevant and meaningful parameter to evaluate and report because they can further reinforce the value of an intervention from a patient and societal perspective, and can justify social funding for interventions that deliver societal value. The WPAI is a PRO tool that can assess productivity losses and general activity impairment but, to our knowledge, has not been previously validated in CF. Our findings show that school productivity loss was significantly lower and activity impairment was numerically lower in the *G551D*/IVA group than in the *F508del*/SOC group. While these results are encouraging, they are based on a subgroup of the study population and further research including a larger group of patients is needed.

Several limitations of this cross-sectional, observational study should be noted. The outcomes assessed were compared in patients with different genotypes. There has been debate in the literature regarding whether patients with class III (e.g. *G551D*) genotypes have disease severity similar to that of patients with class II (e.g. *F508del*) genotypes. Whereas some studies have shown less severe disease in patients with class III than with class II genotypes, others have found these genotypes to be generally comparable [25–29], although variation in the size of the studies may account for some of the differences reported. The observational nature of the study does not allow for the differences in HRQoL between groups to be directly attributed to IVA, in particular because only one group was exposed to this therapy. For patients in the *F508del*/SOC group, the exact nature of SOC was not defined, nor were potential differences in the components of what is regarded as SOC in different countries taken into account.

In our sample, we observed differences between groups, such as the *G551D*/IVA group having a higher proportion of female patients, higher ppFEV_1_ values, and fewer comorbidities than the *F508del*/SOC group. These differences may relate to the fact that the patients within the *G551D*/IVA group were treated with IVA. The significantly lower rate of pancreatic insufficiency seen in the *G551D*/IVA group, for example, may be associated with IVA treatment, since patient clinical characteristics were collected at the time of the survey when patients were long established on therapy. Furthermore, 3 patients with lung transplants were included in the *F508del*/SOC group. Ideally, such patients should have been excluded by the study protocol. However, as they constitute only 2.3% of the *F508del*/SOC group, and their inclusion likely favors the *F508del*/SOC group, we do not envisage that their exclusion would have substantially changed the results. In addition, excluding these patients may favor the results of the *G551D*/IVA group. Our analysis controlled for differences in patient demographic and clinical characteristics between groups; nevertheless, underlying differences between genotypes that could not be accounted for may still exist. In addition, this was an international multicenter study; therefore, available treatment options and corresponding patient burden may vary across different countries/sites [30]. Furthermore, we saw some imbalances in recruitment across different countries, which differed by group. Although systematic recruitment was attempted, data may reflect patients only in the centers studied, and, as such, these results may not be fully generalizable. As with any cross-sectional, observational study, the data do not capture change over time or treatment effects. However, they do highlight the importance of assessing PROs longitudinally in patients, before and during treatment with a new CFTR modulator therapy. With the growing importance of PROs as a measure of the benefit of therapy from the patients’ own perspectives, we would suggest that future longitudinal trials of CFTR modulators in patients with CF should include relevant PROs in their study design. Although resource constraints make routine administration of the CFQ-R impractical in the real-world clinic setting, the simplicity and low costs associated with the EQ-5D-5L confer potential for more widespread use and capture of longitudinal data.

## Conclusions

In summary, patients with CF and a *G551D-CFTR* mutation on ≥1 allele and receiving IVA in real-world settings reported better generic and disease-specific HRQoL, better school productivity, and better symptomatology than patients with CF who are homozygous for *F508del-CFTR* and receiving SOC (without LUM/IVA) in multiple domains. These data support further longitudinal, multicenter assessments to continue to investigate real-world benefits with IVA in patients with CF and a *G551D-CFTR* mutation.

## Data Availability

The datasets used and/or analyzed during the current study are available from the corresponding author on reasonable request.

## Abbreviations

BMI: Body mass index
CF: Cystic fibrosis
CFQ-R: Cystic Fibrosis Questionnaire-Revised
CFTR: Cystic fibrosis transmembrane conductance regulator
EQ-5D-5L: EuroQol 5-dimensions 5-level questionnaire
HRQoL: Health-related quality of life
HTA: Health technology assessment
IVA: Ivacaftor
LS: Least squares
LUM: Lumacaftor
ppFEV_1_: Percent predicted forced expiratory volume in 1 s
PRO: Patient-reported outcome
SD: Standard deviation
SE: Standard error
SOC: Standard of care
VAS: Visual analog scale
WPAI: Work Productivity and Activity Impairment Questionnaire
